# Malignancy estimation of Lung-RADS criteria for subsolid nodules on CT: accuracy of low and high risk spectrum when using NLST nodules

**DOI:** 10.1007/s00330-017-4842-8

**Published:** 2017-04-24

**Authors:** Kaman Chung, Colin Jacobs, Ernst T. Scholten, Onno M. Mets, Irma Dekker, Mathias Prokop, Bram van Ginneken, Cornelia M. Schaefer-Prokop

**Affiliations:** 1 Diagnostic Image Analysis Group, Department of Radiology and Nuclear Medicine, Radboud University Medical Centre, Geert Grooteplein 10, 6525 GA Nijmegen, The Netherlands; 2 Department of Radiology, University Medical Center Utrecht, Utrecht, The Netherlands; 3 Department of Radiology, Meander Medical Center, Amersfoort, The Netherlands

**Keywords:** Subsolid, Pulmonary nodules, Lung cancer, Screening, Management

## Abstract

**Purpose:**

Lung-RADS proposes malignancy probabilities for categories 2 (<1%) and 4B (>15%). The purpose of this study was to quantify and compare malignancy rates for Lung-RADS 2 and 4B subsolid nodules (SSNs) on a nodule base.

**Methods:**

We identified all baseline SSNs eligible for Lung-RADS 2 and 4B in the National Lung Screening Trial (NLST) database. Solid cores and nodule locations were annotated using in-house software. Malignant SSNs were identified by an experienced radiologist using NLST information. Malignancy rates and percentages of persistence were calculated.

**Results:**

Of the Lung-RADS 2SSNs, 94.3% (1790/1897) could be located on chest CTs. Likewise, 95.1% (331/348) of part-solid nodules ≥6 mm in diameter could be located. Of these, 120 had a solid core ≥8 mm, corresponding to category 4B. Category 2 SSNs showed a malignancy rate of 2.5%, exceeding slightly the proposed rate of <1%. Category 4B SSNs showed a malignancy rate of 23.9%. In both categories one third of benign lesions were transient.

**Conclusion:**

Malignancy probabilities for Lung-RADS 2 and 4B generally match malignancy rates in SSNs. An option to include also category 2 SSNs for upgrade to 4X designed for suspicious nodules might be useful in the future. Integration of short-term follow-up to confirm persistence would prevent unnecessary invasive work-up in 4B SSNs.

***Key points*:**

*• Malignancy probabilities for Lung-RADS 2/4B generally match malignancy risks in SSNs.*

*• Transient rate between low-risk Lung-RADS 2 and high-risk 4B lesions were similar.*

*• Upgrade of highly suspicious Lung-RADS 2 SSNs to Lung-RADS 4X might be useful.*

*• Up to one third of the benign high-risk Lung-RADS 4B lesions were transient.*

*• Short-term follow-up confirming persistence would avoid unnecessary invasive work-up of 4B lesions.*

## Introduction

Pulmonary subsolid nodules (SSNs) are a distinctive from solid nodules with respect to CT morphology and underlying pathology [1]. Among the group of SSNs, nonsolid (pure ground-glass) nodules are differentiated from part-solid nodules dependent on the presence of a solid component. In adenocarcinomas, the size of the solid component correlates with the presence of an invasive component on pathology, which has therapeutic and prognostic implications [2, 3]. Non-solid nodules are commonly associated with having a more indolent behavior compared to part-solid nodules in terms of doubling time and malignancy rate [4–7]. It is thus not surprising that management recommendations make a distinction between part-solid and nonsolid nodules [1, 8, 9]. Similarly, as solid nodules, SSNs do not always present as malignancies. SSNs are frequently caused by benign disease, e.g. infection, focal fibrosis, and organizing pneumonia [10–13]. Some guidelines therefore, recommend a 3-month follow-up CT to check lesion persistence for nonsolid nodules larger than 5 mm and part-solid nodules of all sizes [1, 14].

The Lung Imaging Reporting and Data System (Lung-RADS™) was published in 2014 by the American College of Radiology (ACR) to standardize interpretation of screen-detected nodules and harmonize nodule management [8]. It consists of numerous categories depending on nodule type and diameter thresholds. Nodule management is adapted to the relative risk of the nodule to represent or develop into a malignancy. A summary of the Lung-RADS categories can be found in Table 1.

**Table 1:** Summary of Lung-RADS category 1 to 4X

Category Descriptor	Category	Malignancy probability	Management recommendation
Negative	1	<1%	Continue annual screening with LDCT in 12 months
Benign appearance or behavior	2	<1%	Continue annual screening with LDCT in 12 months
Probably benign	3	1-2%	6-month LDCT
Suspicious	4A	5-15%	3-month LDCT; PET/CT may be used when there is a ≥ 8 mm solid component
	4B	>15%	Chest CT with/without contrast, PET/CT and/or tissue sampling depending on the probability of malignancy and comorbidities. PET-CT may be used when there is a ≥ 8 mm solid component
	4X		

Nonsolid nodules are in Lung-RADS category 2 if the diameter is <20 mm. Nodules of that category are considered to have a “benign appearance or behavior” with a malignancy probability of <1% [8]. Though known for their indolent behavior, studies have shown that nonsolid nodules may actually represent invasive adenocarcinomas, especially when the lesion is larger than 10 mm [15, 16]. This makes Lung-RADS category 2 susceptible to underestimation of the malignancy risk. At the other end of the risk spectrum are category 4B nodules, which represent the highest risk group with a malignancy probability score >15%. They are characterized by a solid core of ≥8 mm and management recommendations for these lesions include a clinical chest CT, PET/CT, or a biopsy. Opposed to other guidelines [1, 14], Lung-RADS does not recommend a general 3-month low-dose follow-up for part-solid nodules to confirm persistence. Only category 3 part-solid nodules are recommended to have a 3-month follow-up. This makes category 4B lesions susceptible to overestimation of the malignancy risk because of the morphological overlap with transient infectious nodules that might receive unnecessary invasive diagnostics. Given the considerable management consequences, we were interested to investigate whether these two Lung-RADS categories reflect the proper malignancy risk and consequently appropriate management recommendations for SSNs.

For these reasons, we applied Lung-RADS categories 2 and 4B to SSNs of the largest publicly available database of the National Lung Screening Trial (NLST). The purpose was to quantify the actual malignancy rates on a nodule base and compare these to the malignancy probabilities given by Lung-RADS.

## Materials and methods

### Study group and nodule annotations

Chest CT scans from the NLST were used in this study [17]. All chest CT scans with at least one SSN annotated by the NLST were analyzed, resulting in 3185 participants. The NLST was approved by the institutional board at each participating medical institution and participants provided written informed consent before randomization [17]. CT data, demographics, and information on nodule classification, location, and histology were made available from the NLST after approval of our project proposal.

We selected the type of the SSN according to the NLST database. Longitudinal information about each SSN was needed, as well as which nodule resulted in lung cancer. However, the NLST did not use unique lesion identifications for nodules along the timeline, impeding determination of lesion persistence. Likewise, no information about solid core size was available in the NLST database. Thus, all eligible lesions were reannotated by two medical students and a researcher with in-house software that uses unique lesion identifications for each nodule (CIRRUS Lung Screening, Diagnostic Image Analysis Group, Nijmegen, the Netherlands). Lobe location, size, nodule type, and section numbers stored in the NLST database were used to annotate and characterize each positive nodule (≥4 mm). Nodule classification as nonsolid or part-solid was adopted from the NLST database. Lesion annotation was done on baseline and follow-up CT scans. Lung-RADS categorization was done for nodules on baseline scans and only this data was subsequently analyzed. Information of follow-up scans was used to determine persistence and growth of SSNs to identify malignancies and infectious lesions.

### Identification of malignancies on nodule base

NLST provides information on cancer diagnoses on participant level, but no data are available on which nodular lesion indeed represented the actual malignancy. Therefore, an experienced radiologist (ETS) identified those nodules that were most likely to be the malignancies. The procedure was aided by the anatomic information from the NLST pathology database. Since only the pulmonary lobe where the malignancy had been located and the year of diagnosis were provided by the NLST database, a scale from 0 to 4 was designed to code the likelihood of correct malignancy identification:

0 = *No lesion visible in the tumour lobe at baseline.*

1 = *Lesion highly unlikely to represent cancer.*

2 = *No decision possible.*

(Lesion located in the tumour-bearing lobe that could potentially develop into a malignancy over time, but diagnosis was made >1 year apart of the last available screening CT and available imaging did not reveal unequivocal signs of malignancy.)

3 = *Lesion highly likely to represent cancer.*

(Lesion located in the tumour-bearing lobe and imaging signs very suggestive for malignancy; however, diagnosis of malignancy was made >1 year apart of the last available screening CT.)

4 = *Cancer.*

(Lesion located in the tumour-bearing lobe on a screening CT obtained in the year of tumour diagnosis and imaging signs very suggestive for malignancy).

Imaging signs suggestive for malignancy included spiculation of the solid component and/or evident growth on follow-up.

Only lesions with scores 3 and 4 were included in the data analysis. All SSNs not indicated as being diagnosed as malignancies within the median 6.5 years of follow-up according to the NLST database were considered benign.

### Statistics

Malignancy rates, defined as number of malignancies divided by the total number of lesions in this category multiplied by 100, were calculated for both categories 2 and 4B. Impact of variable size thresholds were calculated for nonsolid nodules in category 2. Percentages of transient and persistent lesions were calculated for both Lung-RADS categories.

## Results

### Study population

The NLST database lists in total 1897 Lung-RADS 2 SSNs. This set consists of 1742 nonsolid nodules <20 mm and 155 part-solid nodules <6 mm. Of these nodules described in the NLST database, 94.4% (1644/1742) of the nonsolid and 94.2% (146/155) of the part-solid nodules could be securely located on the CT images, and thus 1790 Lung-RADS 2 SSNs were eligible for inclusion in this study. Reasons for lack of identification were incomplete DICOM data (N=41) or the fact that a lesion could not be found on the scan (N=66).

Likewise, the NLST database lists 348 part-solid nodules ≥6 mm. Of these part-solid nodules ≥6 mm, 331/348 (95.1%) could be located on the scans. Among this group, 120 lesions had a solid core ≥8 mm, corresponding to Lung-RADS 4B. Reasons for lack of identification were incomplete DICOM data (N=7) or the lesion could not be found on the scan (N=10).

Ten nodules (N=7 in Lung-RADS 2, N=3 in Lung-RADS 4B) had to be excluded in participants with score 0, 1, or 2 assigned during the malignancy identification process. Thus, the final study group included in the statistical analysis consisted of 1783 Lung-RADS 2 and 117 Lung-RADS 4B nodules.

### Malignant nodules in Lung-RADS category 2

**Fig. 1:**
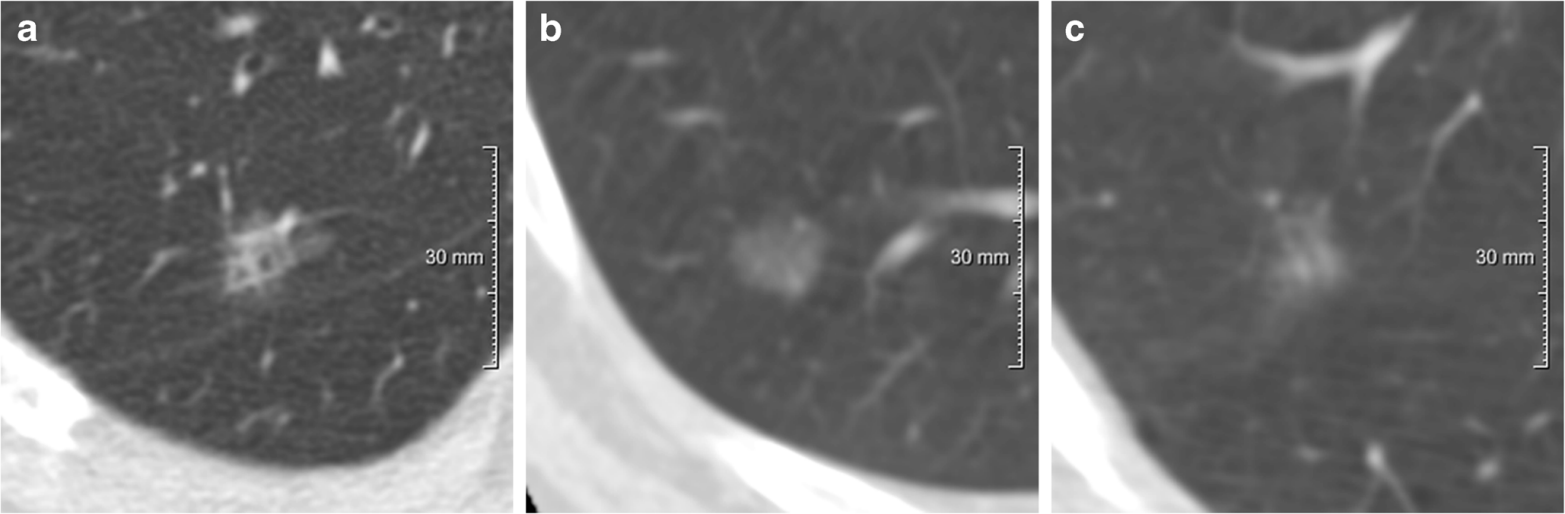
Examples of malignant Lung-RADS category 2 subsolid nodules between 10–15 mm

Forty-four of the Lung-RADS 2 lesions (2.5%; 44/1783) were found to be malignant (Table 2). The malignant lesions had an average diameter of 9.9 mm. The vast majority were nonsolid at baseline (N=42), the remaining two were part-solid. At baseline 20 lesions were <10 mm in diameter, 20 between 10 and 15 mm, and 4 between 15 and 20 mm. Eight lesions were resected within 1 year of baseline, 13 in year 1 after baseline, 11 in year 2 after baseline, and 12 in year 3 after baseline or later. Figure 1 shows examples of malignant category 2 lesions.

**Table 2:** Malignancy rate of Lung-RADS category 2 subsolid nodules

	Lung-RADS category 2 (N = 1783*)
**Malignant**	**44 (2.5%)**
*Nonsolid*	*42*
*Part-solid*	*2*
**Benign**	**1739 (97.6%)**
**No follow-up scan**	**129 (7.4%)**
*nonsolid*	*122*
*part-solid*	*7*
**Transient**	**523 (30.1%)**
*nonsolid*	*489*
*part-solid*	*34*
**Persistent**	**1087 (62.5%)**
*nonsolid*	*984*
*part-solid*	*103*

Within the category 2 group of benign lesions both transient and persistent subsolid nodules can be found and are listed per nodule type. Persistence could not be determined in lesions with no follow-up scan available.

* excluding nodules with malignancy probability scores 0, 1, and 2 (N = 7 lesions)

Of the benign Lung-RADS 2 lesions, 30.1% (523/1740) were found to be transient. Thirty-four were part-solid and 489 were nonsolid. Of the transient nonsolid nodules at baseline 391 were <10 mm in diameter, 83 between 10 and 15 mm, 15 between 15 and 20 mm.

### Impact of varying diameter thresholds on management of Lung-RADS 2 nonsolid lesions

Lowering the threshold from 20 to 15 mm, would have upgraded four of the 42 nonsolid malignancies to a higher Lung-RADS category—and thus earlier follow-up (6 months)—at the expense of earlier follow-up scans for 49 benign nonsolid nodules. Accordingly, lowering the threshold from 20 to 10 mm, would have correctly upgraded 24 malignant nodules to an earlier follow-up but at the expense of earlier follow-up scans for 262 benign nonsolid nodules.

### Benign Nodules in Lung-RADS category 4B

**Fig. 2:**
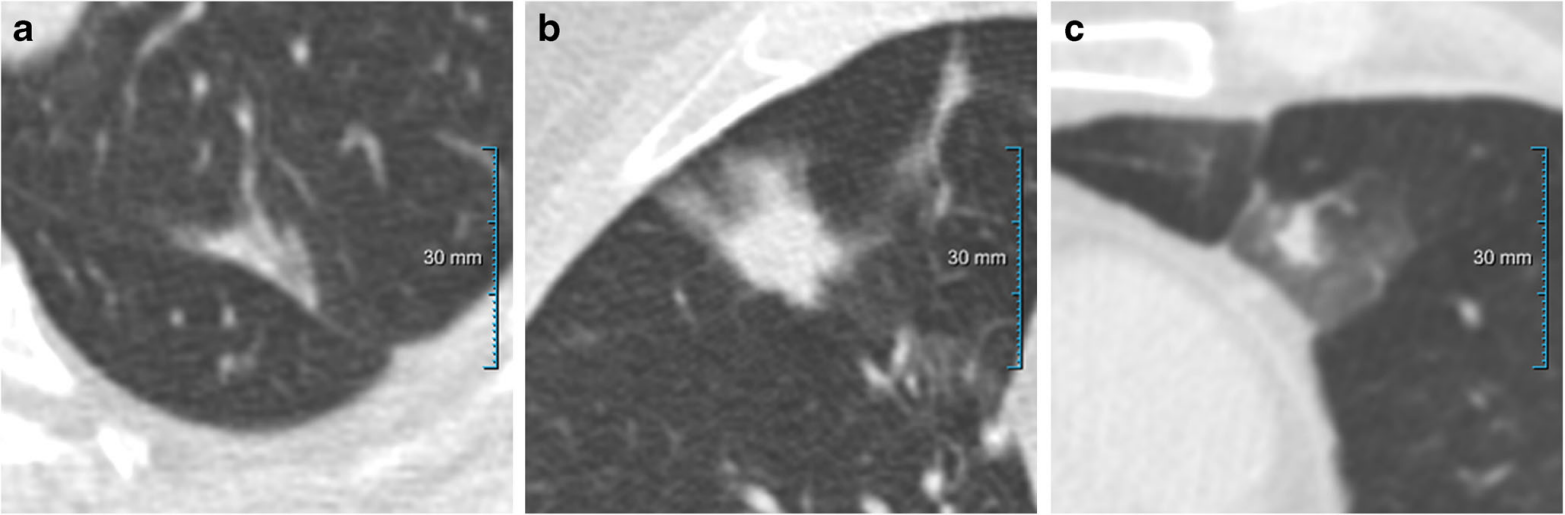
Examples of benign Lung-RADs 4B subsolid nodules, (a, b) persistent (c) transient

In this subset of nodules, 28/117 (23.9%) lesions were found to be malignant (Table 3). The remaining 89/117 (76.1%) lesions were found to be benign. Malignant 4B lesions had an average diameter of 18.0 mm. Eighteen lesions were resected within one year of baseline, six in year 1 after baseline, one in year 2 after baseline, and three in years 3 after baseline or later. Twenty-seven out of the 89 (30.3%) benign lesions were transient and were no longer present on the follow-up screening CT scan. Six of the benign 89 nodules had no follow-up scan to confirm or exclude persistence. Figure 2 shows examples of transient and persistent category 4B lesions.

**Table 3:** Malignancy rate of Lung-RADS category 4B subsolid nodules

	Lung-RADS category 4B (N = 117*)
**Malignant**	**28 (23.9%)**
**Benign**	**89 (76.1%)**
*No follow up scan*	*6 (6.7%)*
*Transient*	*27 (30.3%)*
*Persistent*	*56 (62.9%)*

Within the category 4B group of benign lesions both transient and persistent subsolid nodules can be found. Persistence could not be determined in the lesions with no follow-up scan available.

* excluding nodules with malignancy probability scores 0, 1, and 2 (N = 3 lesions)

## Discussion

In 2014, the ACR published Lung-RADS for screen-detected pulmonary nodules. Similarly to other management recommendations, it differentiates nodule types and uses nodule diameter and growth as major input parameters [1, 8, 14, 18]. Nodule management is adapted to estimated malignancy probability with category 2 corresponding to the finding of a nodule with benign appearance and very low risk of being a malignancy, and category 4B corresponding to the finding of a suspicious nodule with a high malignancy risk, requiring more intense diagnostic work-up including biopsy or resection. In Lung-RADS, thresholds for nodule diameters and corresponding risk estimates had been determined by a consensus panel of experts and were based on publications of data on a summary-level of various screening trials including NLST, ELCAP, and NELSON [19]. Recently, Pinsky et al. [19] published a retrospective analysis of the Lung-RADS performance in the NLST. Lung-RADS scores 1 and 2 were defined as negative and scores 3 to 4B as positive screening results. Performance was analyzed on participant level with respect to the presence of diagnosed malignancy. However, no individual nodules were tracked and no differentiation was made between part-solid nodules with various sizes of solid components (categories 3-4A-4B), since this information is not automatically available by the NLST-database. The fact that a subset of nonsolid nodules <20 mm in diameter is malignant was well discussed, but no further information on distribution of diameter with respect to histology was provided. We, therefore, focused our analysis on the two Lung-RADS categories 2 and 4B, the first with respect to the presence of malignancies despite of low risk categorization, the latter because of its potential overcall of transient infectious lesions as high-risk nodules triggering substantial management consequences.

In our study, we found a malignancy rate of 2.5% in Lung-RADS 2 subsolid lesions and 23.9% for Lung-RADS 4B subsolid lesions. Malignancy-rates published by Lung-RADS that include all nodule types are <1% for Lung-RADS 2 and > 15% for Lung-RADS 4B.

Lung-RADS chose for a relatively large cut-off of 20 mm for nonsolid nodules in the lowest risk category. Both the Fleischner Society and, if there is no previous imaging, also the British Thoracic Society, recommend an initial 3-month follow-up for nonsolid lesions ≥5 mm to assess persistence. The NCCN further differentiates between nodules <5 mm, 5–10 mm, and larger and propose a generally closer follow-up for nonsolid nodules: ≤5 mm yearly LDCT, and for lesions >5-10 mm an LDCT in 6 months, and for lesions >10 mm an LDCT in 3–6 months. Uniformly, all guidelines recommend more invasive diagnostics in subsolid nodules with new or growing (solid) components [1, 14, 20]. Pinsky et al. [19] reported in their analysis of NLST data that Lung-RADS underestimates the likelihood of lung cancer in subjects with nonsolid lesions <20 mm or nodules <6 mm. In line with the findings reported by Pinsky et al. [19] our analysis of NLST nodules also showed a slight underestimation of the Lung-RADS 2 probability score in subsolid nodules (2.5% versus <1%).

A recent publication by I-ELCAP reported that nonsolid nodules can be safely monitored with a 1-year follow-up scan [21]. Similarly, Scholten et al. [22] found that long-term follow-up appears to be safe to check for changes in SSNs. There are, however, studies that have reported a non-negligible percentage of nonsolid lesions representing invasive carcinomas [15; 16]. Hence, it is not yet clarified whether indeed all nonsolid nodules represent indolent malignancies or whether other texture features such as border characteristics or spatial attenuation characteristics beyond diameter or lack of solid component need to be considered to correctly assess malignancy risk [23, 24]. Part of this controversy is most likely related to the variability of differentiating nonsolid from part-solid nodules [25, 26]. A general lowering of the threshold to 15 or 10 mm seems not advisable given the disadvantage of inducing large numbers of false-positive follow-ups and the fact that I-ELCAP could not observe a stage shift/deterioration of patient outcome applying a 1-year follow-up strategy [21]. Interestingly 18% (8/44) of category 2 malignancies were resected within 1 year of the screening CT, another 55% (24/44) within the following 2 screening years, supporting the idea that—although classified as category 2—they displayed some morphological features that made them more suspicious. In addition to the size and nodule-type based categories Lung-RADS includes an additional category 4X triggering more intense diagnostic work up. This category is meant for categories 3, 4A, and 4B nodules that based on visual assessment of the radiologist demonstrate suspicious malignant imaging features justifying an upgrade to a more intense work-up. Currently, category 2 lesions are not included in such a procedure. Therefore, instead of lowering diameter thresholds, it could be an option to also allow for a subset of category 2 lesions an upgrade to category 4X, when radiologists find suspicious malignant imaging features. In that context it is noteworthy that according to Pinsky et al. [19] proportion of stage 1 cancers and 5-year lung cancer-specific survival were not found to be significantly different between malignancies in Lung-RADS category 2 and malignancies in Lung-RADS categories 3, 4A, or 4B subjects, indicating that category 2 malignancies cannot all be considered as indolent [19]. Their results are thus at least partially controversial to the findings published by Yankelevitz et al. [21]. It has to be noted that we do not know how many of the Lung-RADS 2 subsolid lesions in our study group represented adenocarcinoma in situ (AIS) minimally invasive adenocarcinoma (MIA) or indeed invasive adenocarcinomas. AIS and MIA have a (near) 100% disease-free survival after resection and usually grow slowly [27]. Several studies have investigated which morphological features would allow for differentiating pre-invasive from invasive nonsolid lesions [15, 28]. More research would be beneficial to reveal more insight in predictive morphological features of pre-invasive and invasive nonsolid nodules.

Quantification of the potential overestimation of category 4B SSNs was the other purpose of our study. This phenomenon actually has a larger impact on patient management, since category 4B results in a more intense and potentially invasive work-up including a “clinical chest CT, PET-CT, and/or tissue sampling depending on the probability of malignancy, comorbidities, and the radiologist’s final decision” [8].

Lung-RADS published an overall malignancy rate for all nodule types of >15% for category 4B, which is in line with the malignancy rate of 23.9% we found in our analysis for SSNs. This indicates that from a statistical point-of-view there is no overestimation. However, more importantly, one third (30.3%) of the benign 4B SSNs were transient as confirmed by follow-up. Although we conclude that the statistical malignancy risk of Lung-RADS 4B SSNs is in agreement with the Lung-RADS guidelines, our results represent a strong argument to include a short-term follow-up after three months to confirm persistency before considering more invasive management strategies, as also has been suggested by other guidelines. The NCCN suggests to perform a LDCT in 3 months to check for growth when there is low suspicion of lung cancer in their highest risk group for part-solid nodules (>8 mm lesion size). As for nonsolid nodules, the BTS recommends a 3-month CT repeat in cases when there is no previous imaging to assess persistence, growth, or change in morphology for part-solid nodules as well. Similarly the Fleischner Guidelines, designed for incidental nodule findings, recommend always to do a short-term at 3 months to confirm persistence in all part-solid nodules [1, 14, 20].

In both categories, we found that about one third of the lesions were transient. This is an important finding because it eliminates any need for further follow-ups. Previous studies report a wide range of numbers for the rate of transient SSNs (ranging between 12-70%) [11, 21, 29–31]. Lee et al. [30] found a rather high rate of 70% of 126 transient part-solid lesions seen in an Asian population. On the other hand, Yankelevitz et al. [21] who analyzed screen-detected nonsolid nodules in a population comparable to the NLST, reported results more similar to our findings. Their study found that 26% (628/2392) of the nonsolid nodules seen at baseline screening subsequently resolved or decreased. The Multicentric Italian Lung Detection trial found similar results with 31% (15/48) of nonsolid nodules to be transient but a lower percentage of 12% (3/26) for part-solid nodules with a solid core <5 mm [31]. Although our finding generally matches previous literature, rate of persistence in SSNs appears to be influenced by type and study group inclusion.

Our study has some limitations. First, the most important limitation is the fact that SSNs not diagnosed as malignancies within a median period of 6.5 years were considered benign though no histological proof is available. However, this assumption seems to be acceptable based on current data knowledge and mirrored by the fact that current guidelines of the Fleischner society recommend a maximum follow-up of SSNs of 5 years [1]. Second, a small subset of SSNs could not be localized as described in the methods section and were excluded. However, this concerned only a small percentage of nodules and therefore it seems unlikely to have substantially influenced results. The NLST does not provide any information which nodule actually represented the resected malignancy. The standard of which nodule was considered malignant was determined by an experienced radiologist having anatomic information and follow-up scans available. To exclude any indeterminate or doubtful lesions a scoring system was defined and only lesions with high confidence scores were included in the final analysis. Third, pathologic subtypes were not available in the NLST database at the time of analysis. Thus, further differentiation between AIS, MIA and invasive adenocarcinomas was not possible. Last, we used the nodule type classification of the NLST-database, which is based on the assessment of the local screening radiologist. Given the observer variability for the assessment of presence and size of the solid core, it is likely that a subset of SSNs might have been classified differently by other observers [25, 26]. Taken together, the limitations might have slightly influenced the percentages described, but are considered unlikely to have any impact on our conclusions.

In conclusion, malignancy probabilities for Lung-RADS 2 and 4B generally match the malignancy risks in SSNs. The slight statistical underestimation of nonsolid nodules appears to be clinically less relevant given the generally indolent character of these lesions and the fact that the management difference only refers to the time interval of low-dose follow-up CT scans. An option to include also category 2 SSNs for possible upgrade to category 4X designed for more suspicious nodules might be considered in the future; however, more research is needed to define which imaging features qualify for such a procedure. Our finding that one third of the benign 4B SSNs were transient and thus falsely categorized as relatively high risk nodules is of significant clinical importance. Integration of a short-term low-dose follow-up to differentiate persistent from transient lesions would avoid unnecessary invasive work-up in Lung-RADS category 4B SSNs, and should, therefore, be considered in future upgrades of Lung-RADS.

## Abbreviations

SSN: Subsolid nodule
NLST: National Lung Screening Trial
Lung-RADS: Lung Imaging Reporting and Data System
ACR: American College of Radiology
CT: Computer Tomography
PET: Positron Emission Tomography
LDCT: Low-dose CT
